# Refined Analysis of Brain Energy Metabolism Using *In Vivo* Dynamic Enrichment of ^13^C Multiplets

**DOI:** 10.1177/1759091416632342

**Published:** 2016-03-07

**Authors:** Masoumeh Dehghani M., Bernard Lanz, João M. N. Duarte, Nicolas Kunz, Rolf Gruetter

**Affiliations:** 1 Laboratory for Functional and Metabolic Imaging (LIFMET), Ecole Polytechnique Fédérale de Lausanne (EPFL), Switzerland; 2 Department of Radiology, University of Lausanne (EPFL), Switzerland; 3 CIBM-AIT, Ecole Polytechnique Fédérale de Lausanne, Switzerland; 4 Department of Radiology, University of Geneva, Switzerland

**Keywords:** ^13^C isotopomers, brain energy metabolism, *in vivo*^13^C NMR spectroscopy, metabolic modeling, neuronal/glial compartment

## Abstract

Carbon-13 nuclear magnetic resonance spectroscopy in combination with the infusion of ^13^C-labeled precursors is a unique approach to study *in vivo* brain energy metabolism. Incorporating the maximum information available from *in vivo* localized ^13^C spectra is of importance to get broader knowledge on cerebral metabolic pathways. Metabolic rates can be quantitatively determined from the rate of ^13^C incorporation into amino acid neurotransmitters such as glutamate and glutamine using suitable mathematical models. The time course of multiplets arising from ^13^C-^13^C coupling between adjacent carbon atoms was expected to provide additional information for metabolic modeling leading to potential improvements in the estimation of metabolic parameters.

The aim of the present study was to extend two-compartment neuronal/glial modeling to include dynamics of ^13^C isotopomers available from fine structure multiplets in ^13^C spectra of glutamate and glutamine measured *in vivo* in rats brain at 14.1 T, termed bonded cumomer approach. Incorporating the labeling time courses of ^13^C multiplets of glutamate and glutamine resulted in elevated precision of the estimated fluxes in rat brain as well as reduced correlations between them.

A powerful approach to study brain metabolism has been the combination of ^13^C-enriched substrates and *in vivo* magnetic resonance spectroscopy (MRS; Gruetter, 2002; Mason and Rothman, 2004; Henry et al., 2006; Lanz et al., 2013b; Rodrigues et al., 2013). Nuclear magnetic resonance (NMR) measurements enable detection of ^13^C label incorporation from infused ^13^C-enriched substrate (e.g., glucose) into different carbon positions of amino acids in brain such as glutamate (Glu), glutamine (Gln), and aspartate (Asp) (de Graaf et al., 2003; Gruetter et al., 2003).

Metabolic modeling of Carbon labeling time courses of amino acids in brain allows quantitative measurements of metabolic fluxes *in vivo* (Gruetter et al., 2001; Rothman et al., 2003; Oz et al., 2004; Lanz et al., 2012). In the past decade, questions have been raised concerning the reliability of some estimated fluxes in brain (Shestov et al., 2007; Uffmann and Gruetter, 2007; Shen et al., 2009) and many efforts have been undertaken to improve their accuracy through adaptation of the metabolic models used to analyze the ^13^C turnover curves. Monte–Carlo simulations showed that an increased number of measured time courses of ^13^C positions of Glu and Gln as input for the metabolic modeling analysis resulted in an improved precision and reliability of brain metabolic fluxes (Shestov et al., 2007). However, the number of measured ^13^C labeling time courses of metabolites relies on the NMR detection method, the chosen magnetic field strength and pulse sequence in different studies, and varied from two to seven ^13^C uptake curves (Mason et al., 1995; Gruetter et al., 2001; Choi et al., 2002; de Graaf et al., 2004; Xin et al., 2010, 2015).

Due to the difficulty of measuring ^13^C multiplets of Glu and Gln *in vivo* with high temporal resolution, derivation of metabolic fluxes has been performed almost exclusively using dynamic positional enrichment (e.g., de Graaf et al., 2003; Gruetter et al., 2003; Henry et al., 2006; Duarte et al., 2011) which is the time course of total ^13^C enrichment of each carbon position of each metabolite.

The measurement of labeling time courses of ^13^C isotopomers noninvasively in the rat brain has been already reported by Henry et al. (2003a). A first analysis of *in vivo* measured ^13^C dynamic labeling of isotopomers in a heart metabolic study led to improved precision of the estimated tricarboxylic acid (TCA) cycle flux (Jeffrey et al. 1999). In a study of brain metabolism by the same group, the ^13^C time courses of the singlet and doublet of cerebral Glu labeled at position C4 was included in the metabolic analysis and resulted in a poorer determination of glutamate/alpha-ketoglutarate and oxaloacetate/aspartate exchange rate, called *V_x_* (Jeffrey et al., 2013), probably reflecting the incomplete description of the Glu C4 singlet turnover by the considered metabolic model.

Recently, a new ^13^C metabolic modeling approach using the concept of bonded cumomers was proposed, taking full advantage of the available time courses of ^13^C multiplets of Glu and Gln in the metabolic model (Shestov et al., 2012). In simulations, the bonded cumomer approach led to better precision than the positional approach in the determination of brain metabolic fluxes using artificial ^13^C time courses of Glu and Gln multiplets generated with the two-compartmental description of brain energy metabolism (Gruetter et al., 2001) for different infused ^13^C-labeled substrates.

Including the individual time courses of ^13^C multiplets in the metabolic modeling process is expected to provide increased number and precision of the metabolic fluxes of interest. Therefore, the aim of this study was to incorporate the ^13^C dynamic labeling of observable multiplets of Glu and Gln measured with *in vivo* ^13^C MRS in the rat brain at 14.1 T to estimate brain metabolic fluxes in the case of a neuronal–glial compartmentalized metabolic network, under infusion of [1,6-^13^C_2_] glucose.

## Methods

### Animal Preparation

All animal procedures were performed according to federal guidelines and were approved by the local ethics committee from the canton Vaud, Switzerland. The presented ^13^C-glucose infusion studies were performed following a previously described protocol (Duarte et al., 2011). Briefly, four male Sprague–Dawley rats (276 ± 11 g, delivered from Charles River Laboratories, France) were fasted for 6 hr prior to the NMR experiment. Animals were intubated and ventilated with 2% isoflurane during surgery. Both femoral veins were catheterized for the administration of glucose and α-chloralose for anesthesia. Two arteries were cannulated for continuous monitoring of physiology (blood pressure and heart rate) and periodic blood sampling for blood gas, plasma lactate, and glucose concentration measurements as well as for further high resolution NMR analyses of ^13^C enrichment of substrates. Body temperature was maintained between 37.0 and 37.5℃ with a temperature-regulated circulating water bath. Following surgery, anesthesia was switched to intravenous α-chloralose administration (bolus of 80 mg/kg and continuous infusion rate of 28 mg/kg/hr). Animals were placed in a home-built holder and the head position was fixed using ear rods and a bite bar.

After adjustment of MRS parameters, an exponentially decaying 5-min bolus of 99%-enriched [1,6-^13^C_2_] glucose (1.1 M in saline solution) was administered. The volume of this bolus was adapted to the basal glycemia in order to reach 70% plasma glucose fractional enrichment (FE) at the end of the 5 min. The bolus was followed by a continuous infusion of 70%-enriched glucose at a rate equivalent to the whole body glucose disposal rate of 33.2 mg/kg/min (Jucker et al., 2002) and adjusted based on periodically measured arterial plasma glucose concentrations in order to maintain a constant glycemia of 350 mg/dl throughout the experiment. This protocol results in a stable 70% plasma glucose FE reached within 5 min (Henry et al., 2002). At the end of experiment, rats were sacrificed and brain extracts prepared as previously described (Duarte et al., 2007) for further processing and analysis.

### In Vivo and In Vitro NMR Spectroscopy

All *in vivo* spectra were acquired on a 14.1 T magnetic resonance imaging system interfaced to a 26-cm horizontal bore magnet (Magnex Scientific, Oxford, UK; Varian, Palo Alto, CA), equipped with 12-cm inner diameter actively shielded gradients reaching 400 mT/m in 120 µs. The coil assembly consisted of a home-built ^1^H quadrature surface coil and an inner ^13^C linearly polarized surface coil.

After initial setting, fast spin echo images (*TR* = 5 s and *TE* = 52 ms; eight echoes) were acquired to select a volume of interests of 320 µl in the brain. Magnetic field homogeneity was adjusted using FAST(EST)MAP (Gruetter and Tkác, 2000). Localized ^1^H NMR spectra were acquired using the spin-echo full-intensity acquired localized spectroscopy (SPECIAL) sequence (echo time of 2.8 ms and repetition time of 4 s). During the labeling experiment, Carbon-13 NMR (^13^C NMR) spectra were acquired *in vivo* using the semi-adiabatic distortionless enhancement by polarization transfer (DEPT) technique combined with 3D-ISIS ^1^H localization (Henry et al., 2003b). Water-soluble metabolites from brain extracts and plasma samples were quantified by ^1^H and ^13^C NMR spectroscopy with a 14.1 T DRX-600 spectrometer equipped with a 5-mm cryoprobe (BrukerBioSpin SA, Fällanden, Switzerland).

### Spectral Analysis

The signal-to-noise ratio (SNR) of acquired ^13^C spectra is an important factor for the accuracy and precision in quantification of individual ^13^C multiplets of Glu and Gln labeled at positions C4, C3, and C2. To achieve higher SNR, a possibility is to increase temporal averaging of ^13^C spectra acquired with 5.3 min time resolution. After correction for phase and frequency drifts, consecutive MRS acquisition blocks were summed two by two resulting in dynamic spectral data with 10.6 min resolution. For every time point, the 10.6-min spectra from four animals were combined to a single data set after correction for frequency drifts. In the averaging process, the possible time shifts between different animals due to intermediate periods of shimming were taken into account. Summed ^13^C NMR *in vivo* spectra were processed using LCModel (Stephen Provencher Inc., Oakville, Ontario, Canada). The basis sets for LCModel were generated using Matlab (TheMathWorks, Natick, MA) by simulating each ^13^C isotopomer with the appropriate chemical-shift and J-coupling pattern as described by Henry et al. (2003b). The dynamically measured ^13^C spectra were scaled based on the Glu pool size measured with ^1^H MRS and the FE of GluC3, which was determined with the following formula: FE (C3) = C4 D34/(C4 S + C4 D34) where C4 D34 indicates area of the C4 doublet resulting from double labeling of Glu at position C4 and C3, and C4 S the singlet area of Glu at position C4. Multiplets of GluC4 resonances were averaged over the last 30 min, assuming steady-state enrichment for GluC4 at this later stage of infusion.

Correction factors, found by *in vitro* ^13^C NMR spectra from brain extracts and standard solutions containing the metabolites of interest (Duarte & Gruetter, 2013), were applied to account for the relative different efficiencies in signal enhancement by polarization transfer in DEPT for the considered carbon positions of Glu and Gln.

### Metabolic Modeling

**Figure 1.**
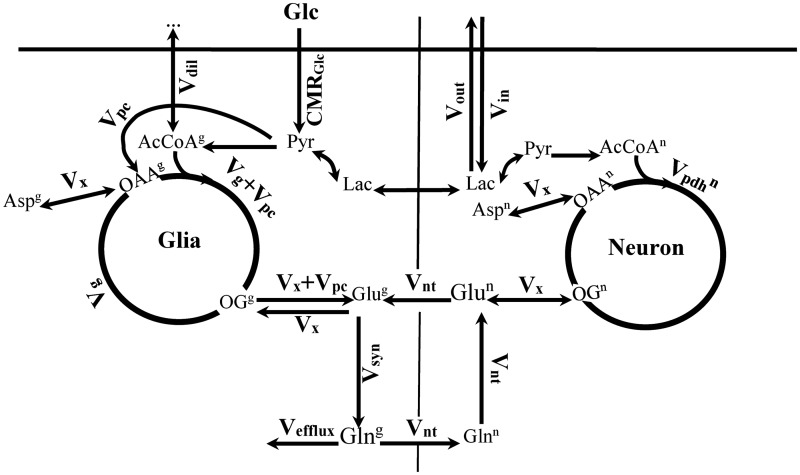
The neuronal–glial compartmentalized metabolic network used for the different metabolic modeling approaches.

The ^13^C turnover curves of Glu and Gln were analyzed using a metabolic model built based on the bonded cumomers concept (Shestov et al., 2012) in the case of a two-compartment neuronal–glial metabolic network description (adapted from Gruetter et al., 2001) shown in Figure 1. This model was adapted to include a nonzero concentration of aspartate in the glial compartment and a dilution factor at the level of glial acetyl-CoA as in our previous study (Duarte et al., 2011). This model introduces and assesses a new metabolic modeling approach that allows the analysis of individual ^13^C multiplets time courses of *in vivo* NMR spectra of the rat brain.

The principles of bonded cumomers modeling were explained comprehensively by Shestov et al. (2012). In summary, a cumomer fraction, noted π*_M_*_{_*_i_*_}_, is the sum of isotopomer fractions for all isotopomers of the molecule M labeled at least at the set of positions $\left\{i\right\}=i_{1},\;i_{2},\ldots ,i_{n}$ independently from the label at other positions. The size *n* of the set {*i*} will be referred to as the order of the π function. Bonded cumomers model consider those cumomers whose indexes refer to adjacent carbons because all possible metabolites isotopomers are not detectable *in vivo* by NMR. As four different splitting patterns are possible for any carbon with two direct carbon neighbors, they can be expressed in terms of the *bonded* ’ bonded cumomers of order *n* ≤ 3.

The following equation shows the transformation for all observable Glu multiplets of the position C4 in a ^13^C spectrum, where the indexes S, *D*, and *Q* refer to singlet, doublet, and doublet of doublet in the GluC4 signal, to the related cumomer fractions:

$$\left(\begin{array}{l} \text{GluC}4\;\text{Q}\\ \text{GluC}4\;\text{D}43\\ \text{GluC}4\;\text{D}45\\ \text{GluC}4\;\text{S} \end{array}\right)=\left(\begin{array}{llll} 0 & 0 & 0 & 1\\ 0 & 1 & 0 & -1\\ 0 & 0 & 0 & -1\\ 1 & -1 & -1 & 1 \end{array}\right)\times \left(\begin{array}{c} {\text{π}}_{\text{Glu}\left\{4\right\}}\\ {\text{π}}_{\text{Glu}\left\{3,4\right\}}\\ {\text{π}}_{\text{Glu}\left\{4,5\right\}}\\ \pi_{\text{Glu}\left\{3,4,5\right\}} \end{array}\right)$$

Furthermore, if the couplings with the two neighboring carbons in the carbon chain of a given metabolite are identical, such as for C3 of Glu, the above equation simplifies to the following transformation, where the index T represents the triplet signal:

$$\left(\begin{array}{c} \text{GluC}3\;\text{T}\\ \text{GluC}3\;\text{D}\\ \text{GluC}3\;\text{S} \end{array}\right)=\left(\begin{array}{llll} 0 & 0 & 0 & 1\\ 0 & 1 & 1 & -2\\ 1 & -1 & -1 & 1 \end{array}\right)\times \left(\begin{array}{l} {\text{π}}_{\text{Glu}\left\{3\right\}}\\ {\text{π}}_{\text{Glu}\left\{2,3\right\}}\\ {\text{π}}_{\text{Glu}\left\{3,4\right\}}\\ {\text{π}}_{\text{Glu}\left\{2,3,4\right\}} \end{array}\right).$$

In the equation above, intensities are normalized so that the total signal is the sum of all multiplet components. For example, in the case of GluC4, we have:

GluC4 tot=GluC4 Q+GluC4 D43 +GluC4 D45+GluC4 S.

The system of differential equations that describes labeling of all observable multiplets of Glu and Gln and the principal chemical intermediates implied in the generation of these multiplets was derived. The final system describing the incorporation of ^13^C from labeled glucose into the multiplets of Glu and Gln consisted in a total of 133 differential equations (see Appendix).

The Cramer–Rao lower bounds (CRLBs) obtained from the LCModel spectral quantification (expressed as coefficient of variation [%standard deviation, *SD*]) were used as a confidence criterion to select the measurable individual ^13^C multiplets of each metabolite. The threshold *SD* to include a multiplet in the metabolic analysis was set to 15% for every observable ^13^C multiplet of Glu or Gln at labeling steady state.

The two-compartment metabolic model was first fitted to the ^13^C enrichment curves of total concentration of Glu and Gln C4, C3, and C2 (i.e., the bonded cumomer model reduced to first-order cumomers, i.e., positional model) over time using the Levenberg–Marquardt algorithm for nonlinear regression, coupled to a Runge–Kutta method for nonstiff systems to obtain numerical solutions of the ordinary differential equations. The different ^13^C uptake curves used in the regression were weighted according to square root of the inverse of the variance (square CRLBs) extracted from the LCModel quantification for every data point of Glu and Gln, to correct for the overweighting of noisy data in the multiple curve regression (Cobelli et al., 2000). The reliability of the estimated fluxes in brain was derived by Monte–Carlo simulation. Artificial data sets (500 series) were generated by adding random Gaussian noise with same variance as the experimental data to each model turnover curve obtained from the best fit of the experimental data. This resulted in 500 data sets with same characteristics as the experimental data but different noise realizations. All numerical procedures were performed in Matlab (MathWorks, Natick, MA).

In a second step, the bonded cumomer model was used for the fitting of the time courses of both the total ^13^C enrichment of every carbon position and every NMR-observable ^13^C multiplet of Glu and Gln which was detected experimentally with sufficient precision (<15% CRLB). The precision of the obtained metabolic fluxes was assessed with an equivalent Monte–Carlo simulation.

To investigate the particular changes in the correlations and probability distributions of *V*_dil_ and *V*_nt_ using both the positional model and bonded cumomer model, a sensitivity analysis was undertaken for the fluxes *V*_dil_ and *V*_nt_. The mentioned fluxes were constrained to a range of chosen values and their effect on the ^13^C labeling curves of Glu and Gln in the positional modeling and bonded cumomer modeling approaches were compared. For each constrained value of *V*_dil_ or *V*_nt_, the metabolic model was fitted to the measured total ^13^C enrichment time courses in positional modeling and total and multiplets ^13^C enrichment time courses in bonded cumomer modeling. In the positional model, the time courses of ^13^C multiplets of Glu and Gln were also simulated in every step of the sensitivity analysis to illustrate the separate effect of *V*_dil_ and *V*_nt_ on the simulated ^13^C multiplets time courses and therefore the potential of adding those curves to the metabolic modeling process. Finally, the effect of removing the time course of total ^13^C enrichment of Glu and Gln C4, C3, and C2 in the bonded cumomer model (referred in following as individual multiplet model) on the precision of the obtained metabolic fluxes was investigated. Statistical significance of differences in flux values found with the various modeling approaches was tested using two-sample unpaired t-test, corrected for multiple comparisons using Bonferroni correction when necessary.

## Results

**Figure 2.**
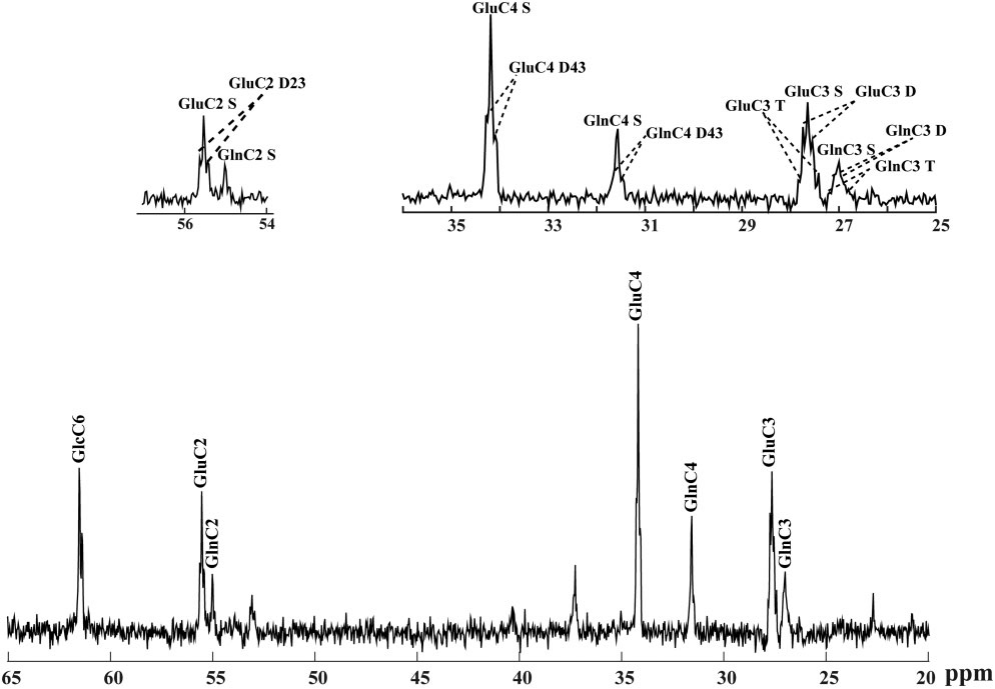
*In vivo* ^13^C NMR spectra acquired three hours after starting [1,6-^13^C_2_] glucose infusion at 14.1 T from a 320 µl volume in the rat brain (summed spectra from four animals with averaged time of 10.6 min, no apodization applied). (b) The fine structure of Glu and Gln at position 4, 3, and 2 is depicted in details in the enlarged spectrum.

To increase the SNR enabling the measurement of isotopomers at low ^13^C enrichment, temporal averaging to a time resolution of 10.6 min of the measured ^13^C MRS data was used and ^13^C spectra from different animals were combined prior to spectral quantification leading to spectra of higher quality and SNR (e.g., SNR of 24 after 2 hr [1,6-^13^C_2_] Glc infusion, based on the GluC4 S peak). The high quality of the spectra was judged from the well-resolved resonances and appearance of multiplet fine structures of Glu and Gln in spatially localized ^1^H-decoupled ^13^C NMR spectra (Figure 2). The total metabolite concentrations measured by ^1^H MRS and averaged over four animals were 8.4 ± 1.2 µmol/g for Glu, 4.3 ± 0.3 µmol/g for Gln, and 2.5 ± 0.3 µmol/g for Asp.

**Figure 3.**
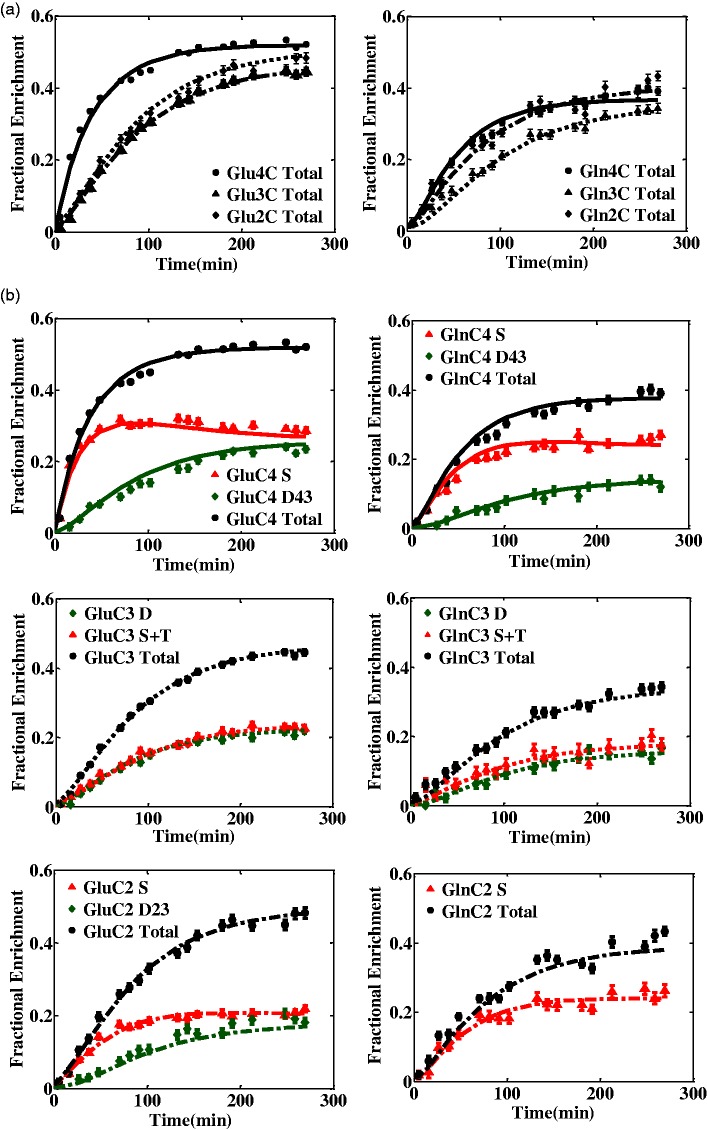
Averaged (four animals) *in vivo* time courses and model fits of Glu and Gln ^13^C enrichment using (a) the positional model and (b) the bonded cumomer model at the carbon positions C4, C3, and C2 during [1,6-^13^C_2_] glucose infusion (each time point averaged over 10.6 min).

The overlap of the central peak of the triplet and the singlet resonances of Glu and Gln labeled at position C3 did not enable their individual spectral quantification, especially at low ^13^C concentrations. A higher estimation of the triplet was consistently associated with an underestimation of the singlet and vice versa. Therefore, the sum of singlet and triplet resonances in GluC3 and GlnC3 time courses (GluC3 S + T and GlnC3 S + T) was considered as input for the cumomer model, taking advantage of the reliable spectral quantification of the summed multiplets GluC3 S + T and GlnC3 S + T, as judged from the respective CRLBs being less than 15% at steady state. The *in vivo* time courses of total ^13^C labeling of Glu and Gln at carbon position C4, C3, and C2 and their observable ^13^C multiplets quantified with *SD* lower than 15% were considered for metabolic modeling analysis during infusion of [1,6-^13^C_2_] glucose (Figure 3). The fitted time courses for the positional model included the total ^13^C enrichment of Glu and Gln at positions C4, C3, and C2. The fitted ^13^C turnover curves for the bonded cumomers approach included the following separate components:

GluC4 total, GluC4 S, GluC4 D43, GluC3 total, GluC3 S + T, GluC3 D, GluC2 total, GluC2 S, GluC2 D23, GlnC4 total, GlnC4 S, GlnC4 D43, GlnC3 total, GlnC3 S + T, GlnC3 D, GlnC2 total, GlnC2 S (total 17 curves). The same notation as Henry et al., (2003a) and Lanz et al. (2013) has been used for the different ^13^C labeling patterns of Glu and Gln.

To determine the metabolic rates upon infusion of ^13^C-enriched glucose, either the positional or the bonded cumomer model was fitted to the aforementioned ^13^C time courses. The resulting metabolic rates (Table 1) included neuronal TCA cycle $\left(V_{\text{pdh}}^{n}\right)$ , glial TCA cycle $\left(V_{\text{TCA}}^{g}=V_{g}+V_{\text{pc}}\right)$ , the malate–aspartate shuttle activity (*V_x_*), apparent neuro transmission flux (*V*_nt_), and glial anaplerotic pyruvate carboxylation (*V*_pc_). Assuming metabolic steady state for the metabolite pools, the cerebral metabolic rate of Glc oxidation (CMR_glc(ox)_), the inflow of labeling from extracerebral lactate (*V*_in_), and the Gln synthesis rate (*V*_syn_) were calculated (Table 1) from the mass balance equations (see A-2). To estimate the pure effect of individual ^13^C multiplets of metabolites as input information for metabolic modeling, brain metabolic fluxes were also estimated by considering all NMR-observable multiplets of Glu and Gln without total ^13^C enrichment time courses (i.e., individual multiplet model) at each carbon position (Table 1). Overall, the estimated metabolic fluxes in all three approaches were precisely determined with *SD* lower than 20% (determined by Monte Carlo analysis) which owes to the good quality of input dynamic ^13^C time courses with adapted time resolution and also the completeness of the used neuronal-glial metabolic model. The most significant differences in the value of estimated fluxes using the different mentioned approaches were for *V*_nt_ and *V*_dil_, with differences of 55% between bonded cumomer model and positional model and 75% between individual multiplet model and positional model.

**Table 1.** The Metabolic Fluxes in µmol/g/min (Value ± *SD*) Determined Using Bonded Cumomer Model, Individual Multiplet Model, and Positional Model.

	Bonded cumomer	Individual multiplet	Positional
Flux (µmol/g/min)			
*V*_g_	0. 17 ± 0.02	0.18 ± 0.03	0.13 ± 0.02
*V_x_*	0.48 ± 0.06	0.57 ± 0.12	0.39 ± 0.06
*V*_nt_	0.063 ± 0.009	0.048 ± 0.007	0.110 ± 0.012
$V_{\mathrm{pdh}}^{n}$	0.33 ± 0.01	0.32 ± 0.01	0.34 ± 0.01
*V*_out_	0.62 ± 0.02	0.67 ± 0.03	0.53 ± 0.04
*V*_pc_	0.040 ± 0.002	0.037 ± 0.003	0.041 ± 0.003
*V*_dil_	0.18 ± 0.02	0.14 ± 0.03	0.31 ± 0.09
Calculated fluxes			
$V_{\mathrm{TCA}}^{g}$	0.21 ± 0.02	0.22 ± 0.03	0.17 ± 0.02
*V*_in_	0.20 ± 0.04	0.24 ± 0.05	0.08 ± 0.05
*V*_syn_	0.10 ± 0.01	0.08 ± 0.01	0.15 ± 0.01
CMR_glc(ox)_	0.29 ± 0.02	0.29 ± 0.03	0.28 ± 0.02

*Note*. The ± *SD* was determined by Monte Carlo analysis over 500 simulations.

**Figure 4.**
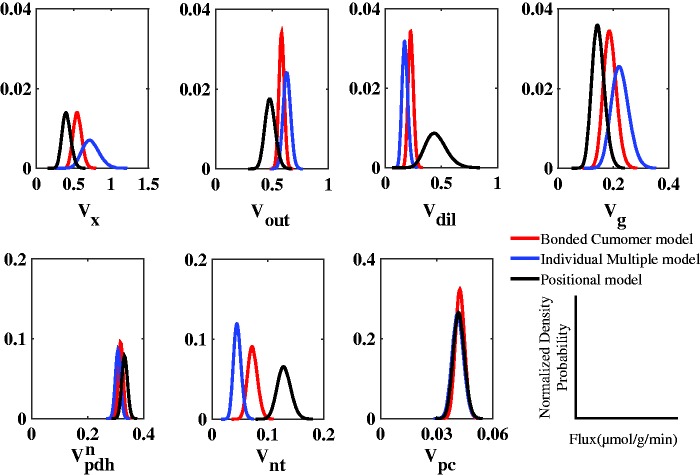
Probability distribution of the determined metabolic fluxes obtained from Monte Carlo simulations (*n* = 500) and fitted with a gamma distribution for visualization purposes, in the cases of the positional model, the individual multiplet model and the bonded cumomer model. The density probability is normalized to the number of simulation, *n* = 500.

The probability distribution of metabolic flux values, calculated by Monte Carlo simulation, was fitted with a gamma distribution for every model (positional model, individual multiplet model, and bonded cumomer model) and compared with each other (Figure 4). The probability density for all estimated fluxes determined by Monte Carlo simulations approached a Gaussian distribution for the three modeling approaches, as a consequence of determined flux values clearly different from zero. In general, the distribution of most metabolic fluxes in every approach was narrow, reflecting the precision of the estimated fluxes and the fact that the models are numerically identifiable. Including ^13^C multiplets time courses of Glu and Gln in the bonded cumomer model resulted in slightly narrower distributions for most metabolic fluxes. The most significant impact was on *V*_dil_ distribution for which the full width at half maximum (FWHM) of the probability distribution decreased by a factor of 5 in the bonded cumomer model compared with the positional model. Removing total enrichment in the individual multiplet model resulted in an approximately twofold increase of the FWHM of most fluxes probability distributions, as compared with the complete bonded cumomer model.

**Figure 5.**
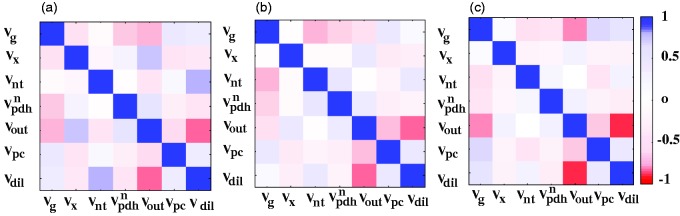
Correlation matrix between metabolic fluxes estimated from the mathematical regression of the turnover curves of Glu and Gln multiplets at position 4, 3, and 2 using (a) positional model, (b) bonded cumomer model, and (c) individual multiplet model.

The effect of the applied modeling approach on the correlation between metabolic fluxes (Figure 5) was an overall decrease of correlations between most metabolic fluxes when applying bonded cumomer modeling. *V*_out_ and *V*_dil_ showed remarkably strong correlations in every approach, more than 80%, independently of the addition of the ^13^C multiplets turnover curves. However, introducing information about time courses of ^13^C multiplets of Glu and Gln had significant impact on reducing correlation between *V*_nt_ and *V*_dil_ in the bonded cumomer model and individual multiplet model, reduced from 80% to 45% and 30%, respectively.

On the other hand, removing information about total ^13^C enrichment of Glu and Gln in the individual multiplet model increased slightly the correlation between several fluxes. For example, the correlation of *V*_dil_ with *V_g_* increased from 13% in bonded cumomer model to 57% in individual multiplet model.

**Figure 6.**
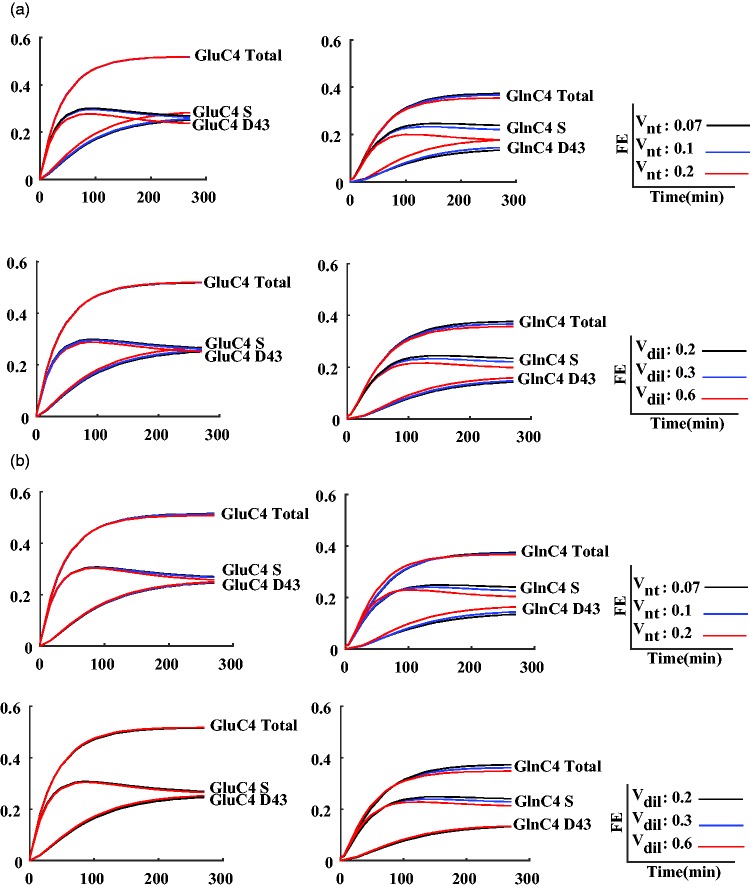
Sensitivity analysis for the fluxes *V*_nt_ and *V*_dil_ plotted for the ^13^C labeling curves of GluC4 and GlnC4 for three different values of *V*_nt_ (0.07, 0.1, and 0.20 µmol g^−1 ^min^−1^) and three different values of *V*_dil_ (0.20, 0.30, and 0.60 µmol g^−1 ^min^−1^) using positional model in the top and bonded cumomer model in the bottom.

In addition, to visualize the improvements obtained with the bonded cumomer model and better understand the particular effect of it on the correlation of *V*_nt_ and *V*_dil_, sensitivity analysis was undertaken for *V*_nt_ and *V*_dil_. In this analysis, the remaining metabolic fluxes were adapted through the nonlinear regression process to get the best fit of the total ^13^C enrichment curves with constrained *V*_nt_ or *V*_dil_. Generally, the time courses of GluC4 and GlnC4 isotopomers showed the highest sensitivity to *V*_nt_ and *V*_dil_, which is illustrated in Figure 6. Total ^13^C enrichment time courses of GlnC4 and GluC4 showed low sensitivity to the constrained value of *V*_nt_ either in the positional or bonded cumomer model. In the positional model, a combination of the remaining fluxes could compensate largely for the fixed *V*_nt_ constraint, except for the steady-state level of GlnC4 total enrichment. On the contrary, the underlying ^13^C multiplet time courses simulated for the positional modeling approach showed a higher sensitivity to changes in *V*_nt_ in particular for GlnC4 doublet and singlet curves. This observation remained valid when including the ^13^C multiplet curves in the fitting process (bonded cumomer model), which shows that no combination of the remaining adjusted parameters enabled to compensate for the fixed *V*_nt_ constraint. ^13^C-labeling curves for doublet and singlet of GlnC4 changed in opposite direction for increasing values of *V*_nt_. As a consequence, the total ^13^C enrichment curve of GlnC4 was not significantly altered. Sensitivity analysis in both models showed that ^13^C time courses of GluC4 isotopomers are not as sensitive as GlnC4 to the value of *V*_dil_. From this analysis, it also appeared that the sensitivity of total GlnC4 ^13^C enrichment to the value of *V*_dil_ was a reflection of GlnC4 singlet sensitivity, while its doublet remained unchanged.

## Discussion

This study demonstrates that using state of the art dynamic ^13^C MRS data under ^13^C-glucose infusion, the recently introduced concept of bonded cumomer metabolic modeling approach provides an extension to the estimation of neuroglial brain metabolic rates, reducing in particular the correlations between most estimated metabolic fluxes in the brain. This kind of approach was applied here for the first time to an extended range of isotopomers and total ^13^C enrichment curves of Glu and Gln (total of 17 uptake curves), quantified from group-averaged *in vivo* ^13^C spectra in rats. The advantages of the bonded cumomer modeling approach and its consistency with previously applied metabolic modeling approaches were evaluated on experimental data.

### Preprocessing Approach for Reliable and Accurate Quantification of Multiplets

Previous simulation studies suggested that the inclusion of the time courses of individual ^13^C multiplets from *in vivo* ^13^C MRS of the brain in the metabolic modeling analysis can result in a significant improvement in the derivation of metabolic fluxes, determined with better precision (Shestov et al., 2012). Direct ^13^C NMR spectroscopy detection at 14.1 T used here benefits from higher SNR and bigger chemical shift dispersion in the NMR signal, potentially allowing higher temporal resolution relative to lower fields (Duarte et al., 2011). The increased chemical shift dispersion of ^13^C resonances allowed to better separate the GluC3 and GlnC3 resonances (Figure 2), which was not possible at lower field such as 9.4 T (Henry et al., 2003b).

However, the detection and quantification of fine structures of ^13^C multiplets by *in vivo* NMR spectroscopy are hampered by inherent effects of homonuclear spin–spin J coupling, which is independent of the applied *B*_0_ magnetic field strength. Therefore, although sensitivity and chemical shift dispersion increase linearly with the applied magnetic field, the spectral separation of the ^13^C multiplets (in ppm) decreases. However, with the higher sensitivity allowed by surface coils, ^1^H decoupling and efficient *B*_0_ shimming using FAST(EST)MAP (Gruetter and Tkác, 2000) method at 14.1 T provided enough homogeneity (less than 10 Hz linewidth) to clearly distinguish the ^13^C multiplets in the spectral fitting.

The total ^13^C enrichment of Glc, Glu, Gln, and Asp were well determined with CRLB lower than 15% at steady state when using a temporal resolution of 5.3 min and SNR of 9 (spectra not shown). However, to overcome the low signal amplitudes of some of the ^13^C multiplets, temporal averaging was increased to 10.6 min and spectra were combined from four animals before spectral quantification, which altogether resulted in a threefold elevation of SNR and better defined ^13^C spectral baseline. This allowed a more robust fitting using LCModel, which in turn resulted in more accurate and reliable quantification of ^13^C multiplets with CRLB less than 15% at steady state (Figure 3).

Significant overlap of the singlet with the center line of the triplet in GluC3 and GlnC3 resulted in high problem of cross correlation and quantification are same for Glu and Gln. Therefore, only the sum of singlet and triplet multiplets was considered for the quantification. The precision on the fit of the sum of singlet and triplet in GluC3 and GlnC3 was systematically better than the precision of the fit when using singlet and triplet components separately.

Overall, the increased resolution and sensitivity at 14.1 T provided ^13^C enrichment turnover curves for GluC4 total, GluC4 S, GluC4 D43, GluC3 total, GluC3 S + T, GluC3 D, GluC2 total, GluC2 S, GluC2 D23, GlnC4 total, GlnC4 S, GlnC4 D43, GlnC3 total, GlnC3 S + T, GlnC3 D, GlnC2 total, GlnC2 S in brain *in vivo*, all of which were with a precision of better than 15% (CRLB) at steady state.

### Positional Model

Nonlinear regression of the positional model to the total measured ^13^C enrichment curves for Glu and Gln at position C4, C3 and C2 resulted in seven independently determined brain metabolic fluxes, without a priori constraints (Table 1). The estimated fluxes and calculated CMR_glc(ox)_ (0.28 ± 0.02 µmol/g/min) were overall comparable with previous ^13^C NMR studies done under α-chloralose anesthesia (for CMR_glc(ox)_: Hyder et al., 1996; 0.18 ± 0.3 µmol/g/min to 0.25 ± 0.03 µmol/g/min; Sibson et al., 1998; 0.26 ± 0.04 µmol/g/min; Duarte et al., 2011; 0.41 ± 0.02 µmol/g/min; Jeffrey et al., 2013; 0.35 ±0.11 µmol/g/min to 0.38 ± 0.12 µmol/g/min; Lanz et al., 2014; 0.41 ± 0.05 µmol/g/min). When quantifying the ^13^C spectra acquired from brain of four animals using the traditional preprocessing approach as in Duarte et al., 2011; namely spectral quantification for individual animals with time resolution of 5.3 min and averaging over the individually quantified data from four animals, the obtained brain metabolic fluxes using time courses of total ^13^C enrichment of Glu and Gln for carbon positions C2, C3, and C4 showed a significantly different *V*_nt_ (*p* < .05, corrected for multiple comparison) as the one estimated in this study (data not shown). The SNR of ^13^C spectra acquired from every individual animal brain with time resolution of 5.3 min (SNR of 9 at steady state) was approximately three times lower than the one obtained by combining the ^13^C spectra from four animals with temporal average of 10.6 min (SNR of 24 at steady state). Not surprisingly, the lower SNR resulted in ^13^C multiplets determined with less precision, which likely reduced the precision of the averaged time courses of ^13^C multiplets. These results illustrate the importance of the spectral preprocessing approach and spectral quality on the estimation of brain metabolic fluxes.

In this study, the exchange rate between cytosolic amino acids and mitochondrial TCA cycle intermediates, *V_x_*, was estimated more precisely (with a relative error lower than 5%) than in previous studies (Choi et al., 2002; Henry et al., 2002). The estimated *V_x_* was slightly higher than the pyruvate dehydrogenase rate, $V_{\text{pdh}}^{n}$ and is consistent with malate–aspartate shuttle being the major mechanism for maintaining the cytosolic redox state under normoxic conditions, as reported before (Gruetter et al., 2001; de Graf et al,. 2004; Yang et al., 2009; Lanz et al., 2014).

In the present work, *V*_nt_ was well determined with a relative *SD* below 10% using positional modeling under [1,6-^13^C_2_] glucose infusion (Table 1). The relative error of all fluxes except *V*_dil_ was lower than 10%, ascribed to the high quality and SNR of the acquired ^13^C spectra at 14.1 T and the refined preprocessing approach used for spectral quantification. Glial labeling dilution of the acetyl-CoA pool, *V*_dil_, had a slightly higher coefficient of variation (19%) and presented strong positive correlation to the apparent rate of neurotransmission, *V*_nt_. This correlation was attributed to the observed difference in ^13^C labeling between Glu C4 and Gln C4. *V*_dil_ creates a difference between the FEs of Glu and Gln while *V*_nt_ is responsible for its dissipation by mixing the two pools.

### Bonded Cumomer Model

The addition of dynamic time courses of ^13^C multiplets of Glu and Gln to the input data for metabolic modeling yielded smaller *SD*s for most fitted metabolic rates compared with positional modeling (Table 1). Probability density functions for brain metabolic fluxes estimated by Monte Carlo simulations (Figure 4) indicated narrower distributions compared with the positional model, consistent with a higher precision of the determined fluxes. One of the largest differences was found in dilution of ^13^C label at the level of glial acetyl-CoA, *V*_dil_, which was more precisely determined in the case of the bonded cumomer model approach.

Using the bonded cumomer model, that is, the inclusion of the dynamic ^13^C labeling of Glu and Gln multiplets in metabolic modeling reduced the mathematical covariance between most fitted fluxes compared with the positional model (Figure 5). The assessment of *V*_dil_ using the positional model resulted in a large covariance between *V*_dil_ and the apparent glutamatergic neurotransmission rate, *V*_nt_. The significantly reduced covariance between *V*_dil_ and *V*_nt_ in the bonded cumomer model is expected to reduce the influence of *V*_dil_ on *V*_nt_ and should allow a more precise and independent measurement of *V*_dil_ and *V*_nt_. Therefore, the distributions of *V*_dil_ and *V*_nt_ determined with the bonded cumomer model are significantly different from ones of the positional model (*p* = .0001 and .01, respectively). *V*_out_ and *V*_dil_ showed remarkably strong correlations in every approach, independently of the addition of the ^13^C multiplet turnover curves of Glu and Gln, which is probably due of the fact that both fluxes contribute to dilute the ^13^C enrichment at the level of acetyl-CoA.

Among the potential reasons for the higher precision and independency of brain metabolic fluxes is the simple increased number of ^13^C time courses and experimental information, arising from dynamic ^13^C enrichment of the individual multiplets of Glu and Gln, as input in the metabolic model, including partially redundant information. However, a more important part is the sensitivity of given ^13^C isotopomers labeling to particular fluxes, which are then averaged out when summing all the isotopomers of the considered carbon position, which is evident in Figure 6 for GlnC4 sensitivity analysis to *V*_nt_ and *V*_dil_.

### Sensitivity Analysis of GluC4 and GlnC4 Multiplets to the Value of *V*_nt_ and *V*_dil_

It is noteworthy that the ^13^C enrichment time courses of total GlnC4 and total GluC4 ^13^C enrichments were less sensitive to *V*_nt_ than the individual ^13^C multiplets of GluC4 and GlnC4; when increasing *V*_nt_, the ^13^C enrichment of the singlet of GluC4 decreased and the ^13^C enrichment of the doublet of GluC4 increased, while the total ^13^C enrichment time course of GluC4 did not change (Figure 6). The remaining metabolic fluxes adapt to the constraints fixed on *V*_nt_ to provide a similar fit quality for total GluC4 in the positional model. This is a manifestation of the higher correlation between *V*_nt_ and most of the remaining fluxes found with the positional modeling approach (Figure 5). For GlnC4, on the other hand, the total ^13^C enrichment curve is sensitive to *V*_nt_ resulting in differences in the ^13^C turnover curve for different constraints on *V*_nt_. Nonetheless, the GlnC4 multiplet curves exhibited also a stronger sensitivity to *V*_nt,_ in particular for GlnC4 S, this already at early time points. As a consequence, including the ^13^C multiplet curves in the cost function of the regression led to a better-defined value of *V*_nt_ in the bonded cumomer model compared with the positional model as well as a lower correlation between *V*_nt_ and most other fluxes in the bonded cumomer model as summarized in Figure 5. Lower correlation of *V*_nt_ with other fluxes in the bonded cumomer model is expected to result in a more independent estimation of *V*_nt_ with less impact from other estimated fluxes.

The above argument was based on the flux *V*_nt_, which shows the strongest changes in terms of correlations with other fluxes between the positional model and the cumomer model. However, note that a general reduction of the correlation coefficients between fluxes is observed with the cumomer model (Figure 5), leading to more precise flux measurements.

Shestov et al. (2007) also showed lower sensitivity of simulated GlnC4 total ^13^C enrichment turnover curve to the value of *V*_nt_ in the positional model. They concluded that this effect was a consequence of the fast ^13^C labeling of the smaller glial Gln pool compared with its indirect precursor, the large neuronal Glu pool. Glial Gln ^13^C labeling therefore is largely influenced by the neuronal Glu pool. However, our results show that changing *V*_nt_ has an effect on the ^13^C turnover curves of GlnC4 multiplets in the bonded cumomer model even though the total ^13^C enrichment of GlnC4 does not change significantly. This can be interpreted in terms of the different precursors of the essentially astrocytic Gln pool: The Gln pool reflects the ^13^C enrichment of the small astrocytic Glu pool, which can be labeled either from the glial TCA cycle or from the neuronal TCA cycle via the neuronal Glu pool. However, the ^13^C labeling patterns in the carbon positions 4 and 3 of Glu generated by the astrocytic or the neuronal TCA cycle are different due to the dilution of the ^13^C labeling position C3 in the glial TCA cycle through pyruvate carboxylase. This can be seen by a lower total ^13^C enrichment in the C3 of Gln as compared with Glu (see Figure 3). The position C4 is also diluted through metabolism of alternative substrates in the astrocytic TCA cycle. Therefore, the probability of generating a doubly labeled C4–C3 Glu molecule from the astrocytic TCA cycle is lower. Increasing the relative value of *V*_nt_ will generate more Gln molecules with carbon backbones originating from the neuronal TCA cycle, with higher double C4–C3 labeling, increasing the doublet component of GlnC4.

For the same reason, the ^13^C labeling time courses of multiplets for Glu and Gln in both models showed that the doublet of GlnC4 is not as sensitive as the singlet of GlnC4 to changes in astrocytic *V*_dil_, representing the glial oxidative metabolism of alternative, unlabeled substrates. Therefore, changes in the total GlnC4 enrichment reflect the sensitivity of GlnC4 S to the value of *V*_dil_ while the ^13^C enrichment curve of GlnC4 D43 does not change significantly.

### Limitation of Modeling Based on ^13^C Labeling Time Courses of Individual Multiplets

Removing total ^13^C enrichment curves from the bonded cumomer model and considering just the ^13^C labeling of individual multiplets had effect on the estimated brain metabolic fluxes in terms of value, precision, and covariance (Figures 4 and 5). The values found for the brain metabolic rates listed in Table 1 were in the range between those found with the positional model and the full-bonded cumomer model. One drawback of excluding the total ^13^C enrichment curves of Glu and Gln in the bonded cumomer model is the reduction of the number of ^13^C time courses as input to the metabolic model. Simulation and experimental studies verified that a higher number of experimental ^13^C time courses of Glu and Gln carbon positions increased the precision of estimated brain metabolic fluxes (Jeffrey et al., 1999; Shestov et al., 2007). In addition, the spectral quantification of the ^13^C multiplet time courses for Glu and Gln has a lower precision due to their lower concentrations when compared with the total ^13^C enrichment.

Using [1,6-^13^C_2_] glucose as a substrate and acquiring ^13^C spectra at a high field of 14.1 T in the present study led to high peak intensities in the ^13^C multiplet resonances of Glu and Gln at position C2, C3, and C4, which made it easier to quantify ^13^C multiplet signals with sufficient sensitivity. However, the ^13^C multiplets are expected to be much more difficult to detect and quantify at lower magnetic fields, in particular when infusing [1-^13^C] glucose and limiting the infusion experiment to 60 to 90 min, as typically undertaken in human studies for cost and practical reasons (Rothman et al., 2011). Therefore, the use of [1-^13^C] glucose remains a limitation for a reliable and accurate quantification of time courses for individual ^13^C multiplets of metabolites.

Early studies on brain extracts (Taylor et al., 1996) proved that coinfusion of [1-^13^C] glucose and [1,2-^13^C_2_] acetate resulted in different ^13^C isotopomer patterns that reflect predominantly neuronal and glial metabolism, respectively. The feasibility of measuring dynamic ^13^C multiplet data in brain arising from simultaneous infusion of [1,6-^13^C_2_] glucose and [1,2-^13^C_2_] acetate *in vivo* has been reported previously (Deelchand et al., 2009). Simultaneous infusion of labeled glucose and acetate is therefore of more interest in terms of amount of information arising from isotopomer ^13^C labeling for brain metabolic modeling. Shestov et al. (2012) showed with simulated data that the best precision in estimating brain metabolic fluxes was obtained in the case of double infusion of [1,6-^13^C_2_] glucose and [1,2-^13^C_2_] acetate. Therefore, future *in vivo* studies with simultaneous infusion of labeled glucose and acetate could take better advantage of dynamic multiplet analysis.

## Conclusion

We conclude that incorporating the ^13^C labeling time courses of multiplets of Glu and Gln measured by ^13^C MRS at high magnetic field (14.1 T) in the neuronal–glial metabolic model brings improvement in the reliability and independency of estimated brain metabolic fluxes. An extended analysis of ^13^C multiplet time courses of metabolites *in vivo* requires infusion of doubly-labeled Glc as substrate and strong *B*_0_ magnetic field in order to get both enough ^13^C enrichment and acceptable SNR in ^13^C multiplets spectra. Therefore, this refined modeling approach may be only applicable for preclinical studies on small animals.

## Appendix

For each multiplet, the index C shows the carbon position in the molecule and the next letter, separated by space, gives the multiplet fine structure (S is for singlet, D for doublet, and T for triplet).

In following equations, the indexes *b* and *p* specify metabolic pools in brain and plasma, respectively. Metabolic pools in the neuronal compartment are indicated with the index *n* and in the glial compartment with the index *g*.

The TCA cycle rate in the neuronal compartment, flux through pyruvate dehydrogenase: $V_{\text{pdh}}^{n}$

The TCA cycle rate in the glial compartment: $V_{\text{TCA}}^{g}\;=\;V_{g}\;+\;V_{\text{pc}}$

Flux through neuronal glutaminase: *V*_nt_

The anaplerotic flux through pyruvate carboxylase: *V*_pc_

Glutamate-alpha–ketoglutarate and oxaloacetate–aspartate exchange rate: *V_x_*

Outflow and inflow of labeling at the level of Lactate: *V*_out_ and *V*_in_

The dilution flux at the level of acetyl-CoA: *V*_dil_

### A-1. Glucose Transport Across the Blood–Brain Barrier

Brain glucose concentration using a reversible Michaelis–Menten kinetics for glucose transport across the blood–brain barrier change according to following expression:

ddt[Glc(t)b]= Tmax[Glc(t)p]−[Glc(t)b]/VdKt+[Glc(t)b]Vd+[Glc(t)p] −CMRGlc.

*T*_max_: The apparent maximal transport rate

*K_t_*: The apparent Michaelis constant for glucose transport

*V*_d_: The physical volume of distribution for glucose in the brain, 0.77 ml/g

### A-2. Mass Balance Equations of Particular Interest

With steady-state assumption for the Glu and Gln labeling pools in the case of [1,6-^13^C_2_] Glc infusion, the total concentration of the labeling pools as well as the metabolic fluxes between the pools be assumed to remain same over the duration of the measurement. Thus, maintaining the mass balance equations results in following expressions (Henry et al., 2006; Lanz et al., 2013)

ddt[Lacb]=Vin−Vout+2CMRglc−Vpdhn−Vg−2Vpc=0.ddt[OAAg]=Vx+Vpc−Vx−Vsyn+Vnt=0.ddt[Glug]=Vpc−Vsyn+Vnt=0·ddt[Glng] =Vsyn−Vefflux−Vnt=0.

The Gln synthesis rate in glial: $V_{\text{syn}}=\;V_{\text{nt}}+V_{\text{pc}}.$

The net Gln efflux: $V_{\text{efflux }}=V_{\text{pc}}.$

The fraction of Glucose metabolized at steady-state in the TCA cycle: ${\text{CMR}}_{\text{glc}(\text{ox})}=\frac{V_{\text{pdh}}^{n}+V_{\text{TCA}}^{g}+V_{\text{pc}}}{2}.$

The cerebral metabolic rate of Glucose consumption: ${\text{CMR}}_{\text{glc}}={\text{CMR}}_{\text{glc}(\text{ox})}+\frac{V_{\text{out}}-V_{\text{in}}}{2}.$

### A-3. Metabolic Equations for Isotopomers Labeling in Bonded Cumomer Model

{*i*}, {*j*}, and {*k*} indicate the positions of labeled carbon in metabolite. ${\text{π}}_{\text{M}\left\{i\right\}}$ refers to the sum of isotopomers fractions labeled at least at position {*i*} and interpreted as the probability of labeling metabolite at position *i*. ${\text{π}}_{\text{M}\left\{i,j\right\}}$ is the probability of metabolite being labeled at adjacent positions *i* and *j*. ${\text{π}}_{\text{M}\left\{i,j,k\right\}}$ indicates the probability metabolite being labeled at three adjacent position *i*, *j*, and *k* (Shestov et al,, 2012).

#### Transport of ^13^C-enriched carbons of glucose

[Glc(t)b]ddt(πGlc{i}b) = Tmax[Glc(t)p](πGlc{i}p)−[Glc(t)b]/Vd(πGlc{i}b)Kt+[Glc(t)b]Vd+[Glc(t)p] −CMRGlc(πGlc{i}b).

[Glc(t)b]ddt(πGlc{i,j}b)= Tmax[Glc(t)p](πGlc{i,j}p)−[Glc(t)b]/Vd(πGlc{i,j}b)Kt+[Glc(t)b]Vd+[Glc(t)p] −CMRGlc(πGlc{i,j}b).

[Glc(t)b]ddt(πGlc{i,j,k}b)= Tmax[Glc(t)p](πGlc{i,j,k}p)−[Glc(t)b]/Vd(πGlc{i,j,k}b)Kt+[Glc(t)b]Vd+[Glc(t)p] −CMRGlc(πGlc{i,j,k}b).

#### Enrichment of neuronal amino acids

[Glun].ddt(πGlu{i}n)=Vx(πOG {i}n)+Vnt(πGln{i}n) −(Vx+Vnt)(πGlu{i}n).

[Glun].ddt(πGlu {i,j}n)=Vx(πOG {i,j}n)+Vnt(πGln {i,j}n) −(Vx+Vnt)(πGlu {i,j}n).

[Glun].ddt(πGlu{i,j,k}n)=Vx(πOG{i,j,k}n)+Vnt(πGln{i,j,k}n)−(Vx+Vnt)(πGlu{i,j,k}n).

$$\left[{\text{Gln}}^{n}\right].\frac{d}{dt}\left({\text{π}}_{\text{Gln}\left\{i\right\}}^{n}\right)=V_{\text{nt}}\left({\text{π}}_{\text{Gln}\left\{i\right\}}^{g}\right)-V_{\text{nt}}\left({\text{π}}_{\text{Gln}\left\{i\right\}}^{n}\right).$$

$$\left[{\text{Gln}}^{n}\right].\frac{d}{dt}\left({\text{π}}_{\text{Gln}\left\{i,j\right\}}^{n}\right)=V_{\text{nt}}\left({\text{π}}_{\text{Gln}\left\{i,j\right\}}^{g}\right)-V_{\text{nt}}\left({\text{π}}_{\text{Gln}\left\{i,j\right\}}^{n}\right).$$

$$\left[{\text{Gln}}^{n}\right].\frac{d}{dt}\left(\begin{array}{c} {\text{π}}_{\text{Gln}\left\{i,j,k\right\}}^{n} \end{array}\right)=V_{\text{nt}}\left(\begin{array}{c} {\text{π}}_{\text{Gln}\left\{i,j,k\right\}}^{g} \end{array}\right)-V_{\text{nt}}\left(\begin{array}{c} {\text{π}}_{\text{Gln}\left\{i,j,k\right\}}^{n} \end{array}\right).$$

$$\left[{\text{Asp}}^{n}\right].\frac{d}{dt}\left({\text{π}}_{\text{Asp}\;\left\{i\right\}}^{n}\right)=V_{x}\left({\text{π}}_{\text{OAA }\left\{i\right\}}^{n}\right)-V_{x}\left({\text{π}}_{\text{Asp}\;\left\{i\right\}}^{n}\right).$$

$$\left[{\text{Asp}}^{n}\right].\frac{d}{dt}\left(\begin{array}{c} {\text{π}}_{\text{Asp}\;\left\{i,j\right\}}^{n} \end{array}\right)=V_{x}\left(\begin{array}{c} {\text{π}}_{\text{OAA}\;\left\{i,j\right\}}^{n} \end{array}\right)-V_{x}\left(\begin{array}{c} \begin{array}{c} {\text{π}}_{\text{Asp}\;\left\{i,j\right\}}^{n} \end{array} \end{array}\right).$$

$$\left[{\text{Asp}}^{n}\right].\frac{d}{dt}\left(\begin{array}{c} \begin{array}{c} {\text{π}}_{\text{Asp}\left\{i,j,k\right\}}^{n} \end{array} \end{array}\right)=V_{x}\left(\begin{array}{c} {\text{π}}_{\text{OAA}\left\{i,j,k\right\}}^{n} \end{array}\right)-V_{x}\left(\begin{array}{c} {\text{π}}_{\text{Asp}\left\{i,j,k\right\}}^{n} \end{array}\right).$$

#### Enrichment of glial amino acids

[Glug].ddt(πGlu{i}g)=(Vx+Vpc)(πOG{i}g)+Vnt(πGlu{i}n) −(Vx+Vnt+Vpc)(πGlu{i}g).

[Glug].ddt(πGlu {i,j}g)=(Vx+Vpc)(πOG {i,j}g)+Vnt(πGlu {i,j}n) −(Vx+Vnt+Vpc)(πGlu {i,j}g).

[Glug].ddt(πGlu{i,j,k}g)=(Vx+Vpc)(πOG{i,j,k}g)+Vnt(πGlu{i,j,k}n) −(Vx+Vnt+Vpc)(πGlu{i,j,k}g).

[Glng].ddt(πGln{i}g)=(Vnt+Vpc)(πGlu{i}g) −(Vnt+Vpc)(πGln{i}g).

[Glng].ddt(πGln{i,j}g)=(Vnt+Vpc)(πGlu {i,j}g) −(Vnt+Vpc)(πGln {i,j}g).

[Glng].ddt(πGln{i,j,k}g)=(Vnt+Vpc)(πGlu{i,j,k}g) −(Vnt+Vpc)(πGln {i,j,k}g).

$$\left[{\text{Asp}}^{g}\right].\frac{d}{dt}\left({\text{π}}_{\text{Asp}\left\{i\right\}}^{g}\right)=V_{x}\left({\text{π}}_{\text{OAA }\left\{i\right\}}^{g}\right)-V_{x}\left({\text{π}}_{\text{Asp}\left\{i\right\}}^{g}\right).$$

$$\left[{\text{Asp}}^{g}\right].\frac{d}{dt}\left(\begin{array}{c} {\text{π}}_{\text{Asp}\left\{i,j\right\}}^{g} \end{array}\right)=V_{x}\left(\begin{array}{c} {\text{π}}_{\text{OAA }\left\{i,j\right\}}^{g} \end{array}\right)-V_{x}\left(\begin{array}{c} {\text{π}}_{\text{Asp}\left\{i,j\right\}}^{g} \end{array}\right).$$

$$\left[{\text{Asp}}^{g}\right].\frac{d}{dt}\left(\begin{array}{c} {\text{π}}_{\text{Asp}\;\left\{i,j,k\right\}}^{g} \end{array}\right)=V_{x}\left(\begin{array}{c} {\text{π}}_{\text{OAA }\left\{i,j,k\right\}}^{g} \end{array}\right)-V_{x}\left(\begin{array}{c} {\text{π}}_{\text{Asp}\;\left\{i,j,k\right\}}^{g} \end{array}\right).$$

#### Enrichment of single brain pyruvate pool by considering its fast equilibrium with lactate

[Pyr].ddt(πpyr {1}πpyr {2}πpyr {3})=CMRGlc(πGlc{3}b+πGlc{4}bπGlc{2}b+πGlc{5}bπGlc{1}b+πGlc{6}b) −(Vout+2Vpc+Vg+Vpdhn)(πpyr {1}πpyr {2}πpyr {3}) +(2Vpc+Vg+Vpdhn+Vout−2CMRGlc)(πLac{1}pπLac{2}pπLac{3}p).

[Pyr].ddt(πpyr {1,2}πpyr {2,3})=CMRGlc(πGlc{2,3}b+πGlc{4,5}bπGlc{1,2}b+πGlc{5,6}b) −(Vout+2Vpc+Vg+Vpdhn)(πpyr {1,2}πpyr {2,3}) +(2Vpc+Vg+Vpdhn+Vout−2CMRGlc)(πLac{1,2}pπLac{2,3}p).

[Pyr].ddt(πpyr {1,2,3})=CMRGlc(πGlc{1,2,3}b+πGlc{4,5,6}b) −(Vout+2Vpc+Vg+Vpdhn)(πpyr{1,2,3}) +(2Vpc+Vg+Vpdhn+Vout−2CMRGlc)(πLac{1,2,3}p).

#### Enrichment of acetyl-CoA

[AcCoAg].ddt(πAcCoA{1}gπAcCoA{2}g)=Vdil(πAce{1}gπAce{2}g)+(Vg+Vpc)(πpyr{2}gπpyr{3}g) −(Vdil+Vg+Vpc)(πAcCoA{1}gπAcCoA{2}g).

[AcCoAg].ddt(πAcCoA{1,2}g)=Vdil(πAce{1,2}g)+(Vg+Vpc)(πpyr {2,3}g) −(Vdil+Vg+Vpc)(πAcCoA{1,2}g).

#### Enrichment of neuronal TCA cycle intermediates

[OGn].ddt(πOG{1}nπOG{2}nπOG{3}nπOG{4}nπOG {5}n)=Vpdhn(πOAA{4}nπOAA{3}nπOAA{2}nπpyr{3}nπpyr {2}n)+Vx(πGlu{1}nπGlu{2}nπGlu{3}nπGlu{4}nπGlu{5}n) −(Vpdhn+Vx)(πOG {1}nπOG {2}nπOG {3}nπOG{4}nπOG {5}n).

[OGn].ddt(πOG {1,2}nπOG {2,3}nπOG {3,4}nπOG {4,5}n)=Vpdhn(πOAA {3,4}nπOAA {2,3}nπOAA{2}nπpyr{3}nπpyr{2,3}n) +Vx(πGlu {1,2}nπGlu{2,3}nπGlu {3,4}nπGlu{4,5}n)−(Vpdhn+Vx)(πOG {1,2}nπOG {2,3}nπOG {3,4}nπOG {4,5}n).

[OGn].ddt(πOG{1,2,3}nπOG{2,3,4}nπOG {3,4,5}n)=Vpdhn(πOAA {2,3,4}nπpyr{3}nπOAA {2,3}nπOAA {2}nπpyr {2,3}n) +Vx(πGlu {1,2,3}nπGlu {2,3,4}nπGlu {3,4,5}n)−(Vpdhn+Vx)(πOG{1,2,3}nπOG{2,3,4}nπOG{3,4,5}n).

[OAAn].ddt(πOAA {1}nπOAA {2}nπOAA {3}nπOAA {4}n)=Vpdhn2(πOG{2}n+πOG{5}nπOG{3}n+πOG{4}nπOG{3}n+πOG{4}nπOG{2}n+πOG {5}n) +Vx(πAsp{1}nπAsp{2}nπAsp{3}nπAsp {4}n)−(Vpdhn+Vx)(πOAA{1}nπOAA{2}nπOAA {3}nπOAA {4}n).

[OAAn].ddt(πOAA {1,2}nπOAA {2,3}nπOAA {3,4}n)=Vpdhn2(πOG {2,3}n+πOG {4,5}n2πOG {3,4}nπOG {2,3}n+πOG {4,5}n) +Vx(πAsp {1,2}nπAsp {2,3}nπAsp {3,4}n)−(Vpdhn+Vx)(πOAA {1,2}nπOAA {2,3}nπOAA {3,4}n).

[OAAn].ddt(πOAA {1,2,3}nπOAA {2,3,4}n)=Vpdhn2(πOG{2,3,4}n+πOG{3,4,5}nπOG {2,3,4}n+πOG{3,4,5}n) +Vx(πAsp {1,2,3}nπAsp {2,3,4}n)−(Vpdhn+Vx)(πOAA{1,2,3}nπOAA{2,3,4}n).

#### Enrichment of glial TCA cycle intermediates

[OGg].ddt(πOG {1}gπOG{2}gπOG{3}gπOG{4}gπOG{5}g)=(Vg+Vpc)(πOAA{4}gπOAA{3}gπOAA{2}gπAcCoA{2}gπAcCoA{1}g)+Vx(πGlu {1}gπGlu{2}gπGlu{3}gπGlu{4}gπGlu{5}g) −(Vg+Vx+Vpc)(πOG{1}gπOG{2}gπOG{3}gπOG {4}gπOG{5}g).

[OGg].ddt(πOG {1,2}gπOG {2,3}gπOG {3,4}gπOG {4,5}g)=(Vg+Vpc)(πOAA {3,4}gπOAA {2,3}gπOAA{2}gπAcCoA{2}gπAcCoA{1,2}g) +Vx(πGlu {1,2}gπGlu {2,3}gπGlu {3,4}gπGlu {4,5}g)−(Vg+Vx+Vpc)(πOG {1,2}gπOG {2,3}gπOG {3,4}gπOG {4,5}g).

[OGg].ddt(πOG{1,2,3}gπOG{2,3,4}gπOG{3,4,5}g)=(Vg+Vpc)(πOAA{2,3,4}gπOAA{2,3}gπAcCoA{2}gπOAA{2}gπAcCoA{1,2}g) +Vx(πGlu{1,2,3}gπGlu{2,3,4}gπGlu{3,4,5}g)−(Vg+Vx+Vpc)(πOG {1,2,3}gπOG {2,3,4}gπOG {3,4,5}g).

[OAAg].ddt(πOAA{1}gπOAA{2}gπOAA{3}gπOAA{4}g)=Vg2(πOG {2}g+πOG {5}gπOG{3}g+πOG{4}gπOG{3}g+πOG{4}gπOG{2}g+πOG{5}g)+Vx(πAsp {1}gπAsp{2}gπAsp{3}gπAsp{4}g) −(Vg+Vx+Vpc)(πOAA{1}gπOAA{2}gπOAA{3}gπOAA{4}g)+Vpc(πpyr{1}gπpyr{2}gπpyr{3}gπCO2).

[OAAg].ddt(πOAA{1,2}gπOAA{2,3}gπOAA{3,4}g)=Vg2(πOG{2,3}g+πOG{4,5}g2πOG{3,4}gπOG{2,3}g+πOG{4,5}g)+Vx(πAsp{1,2}gπAsp{2,3}gπAsp{3,4}g) −(Vg+Vx+Vpc)(πOAA{1,2}gπOAA{2,3}gπOAA{3,4}g)+Vpc(πpyr {1,2}gπpyr {2,3}gπCO2πpyr {3}g).

[OAAg].ddt(πOAA {1,2,3}gπOAA {2,3,4}g)=Vg2(πOG {2,3,4}g+πOG {3,4,5}gπOG {2,3,4}g+πOG {3,4,5}g)+Vx(πAsp {1,2,3}gπAsp {2,3,4}g) −(Vg+Vx+Vpc)(πOAA{1,2,3}gπOAA{2,3,4}g)+Vpc(πpyr {1,2,3}gπpyr {2,3}gπCO2).
